# Risk Factors and Interventional Predictors for Postoperative Malunion and Nonunion in Adult Mandibular Fractures: A Scoping Review

**DOI:** 10.7759/cureus.103931

**Published:** 2026-02-19

**Authors:** Pranav S Tadepalli, Julian Scaccia, Shaan R Patel, Vishnu Vundamati, Harvey N Mayrovitz

**Affiliations:** 1 Medical College, Nova Southeastern University Dr. Kiran C. Patel College of Osteopathic Medicine, Davie, USA; 2 Biology, Nova Southeastern University, Davie, USA; 3 Medical Education, Nova Southeastern University Dr. Kiran C. Patel College of Allopathic Medicine, Davie, USA

**Keywords:** complication, malunion, mandibular fracture, maxillo facial trauma, maxillo-facial trauma, maxillomandibular fixation, nonunion, open reduction internal fixation, risk factor, trauma

## Abstract

Mandibular fractures are one of the most commonly treated injuries in oral and maxillofacial surgery. While malocclusion has been well studied as a postoperative complication, malunion and nonunion remain comparatively unreported despite being significant complications associated with the healing of facial fractures. This scoping review aims to evaluate risk factors, predictors, surgical variables, and management strategies associated with postoperative malunion or nonunion in mandibular fractures. PubMed, Embase, Web of Science, and Google Scholar were searched for English-language studies published between 2000 and 2025. Eligible studies included prospective and retrospective clinical investigations evaluating malunion and nonunion following surgical or nonsurgical management of mandibular fractures. Systematic reviews, meta-analyses, case reports, pediatric studies, and non-English studies were excluded from this review. Extracted data included fracture location and complexity, treatment modality, fixation method, surgical approach, timing of intervention, and reported postoperative outcomes.

Of 1,052 records initially identified, 13 studies met the inclusion criteria. Across study designs, open reduction and internal fixation (ORIF) was consistently associated with lower rates of malunion and nonunion compared with various closed reduction techniques, particularly in comminuted fractures. Delayed surgical intervention, most notably between 6 and 7 days from injury, was associated with increased rates of malunion and nonunion. Additional factors associated with increased complication rates included posterior mandibular fractures, fracture comminution, smoking and alcohol abuse, lack of patient education, and advanced patient age. In conclusion, available evidence suggests that timely ORIF with stable fixation constructs is associated with reduced rates of postoperative malunion/nonunion following mandibular fracture repair. Delayed intervention and inadequate postoperative education protocols are other risk factors and predictors that need to be considered to minimize potential complications.

## Introduction and background

Mandibular fractures are the second most common facial fractures, with approximately 2500 occurring in the United States each year [1]. Young males account for up to 83% of these injuries, likely due to their propensity for involvement in aggressive behaviors, accidents, and violence [2]. Surgical intervention is often employed in the setting of mandibular fractures due to the bone’s complex structural and functional anatomy. The type of management often depends on the extent of trauma [3]. Postoperative complications such as malocclusion, malunion, or nonunion are a significant concern when correcting mandibular fractures, as improper healing may hinder a patient’s recovery process or necessitate repeat interventions.

A conservative closed approach is recommended for uncomplicated mandibular fractures, such as nondisplaced or greenstick fractures. The most common closed technique is maxillomandibular fixation (MMF), in which the upper and lower jaws are wired together to immobilize the bones and facilitate the body’s natural healing process [3]. When this method is not suitable or when multiple fracture fragments are present, open reduction with internal fixation (ORIF) is often employed. ORIF is an open surgical approach in which fracture sites are exposed via small incisions, and various plate fixation methods are used according to the patient's needs [4].

Nonunion and malunion are two postsurgical complications of mandibular fractures that involve the healing of the bone. Nonunion is defined as the body’s inability to heal a fracture for a minimum of nine months, with three months of no signs of healing [5]. It is a multifactorial complication, but it is mainly attributed to the method of intervention. In contrast, malunion is defined as the bone healing in an improper position [6]. Similar to nonunion, it is largely attributed to complications arising from improper intervention techniques. While there is ample research exploring malocclusion, there is little research surrounding malunion/nonunion. If the causes of these complications are understood and addressed, providers can make a more informed decision about which treatment measure to choose to achieve the best results for the patient. This review aims to evaluate risk factors, predictors, and management strategies for postoperative complications of nonunion and malunion following surgical intervention of mandibular fractures.

## Review

Methods

Search Strategy

A comprehensive search was conducted using the following electronic databases: PubMed, Google Scholar, Web of Science, and Embase. The search was conducted using a predefined list of controlled vocabulary terms and keywords. Search terms included: “mandibular fracture,” “malocclusion,” “malunion,” “angle,” “timing,” “intervention,” “risk factors,” and “complications.” These descriptors were applied using the standard Boolean operators (AND, OR, NOT) in accordance with database search engine mechanics to capture as many relevant articles as possible. Full criteria for each database are available in the Appendices.

Eligibility Criteria

Initial data screening was performed by four authors independently, and articles that met the inclusion criteria were included in the preliminary review. Table 1 summarizes the inclusion and exclusion criteria utilized during the initial screening process. 1,052 articles were identified in the initial screening. Utilizing the Rayyan software (Rayyan, Cambridge, USA), 226 duplicates were detected. After eliminating duplicate articles, the authors were left with 826 articles.

**Table 1:** Inclusion and exclusion criteria employed during the search of databases.

Inclusion Criteria	Exclusion Criteria
Studies written in English	Studies published before the year 2000
Peer-reviewed human studies	Animal studies
Any gender	Studies without data on patient risk factors or interventional predictors
Study subject 18 years or older	Study type: systematic reviews, meta-analyses, case reports, gray literature, conference abstracts, editorials, or commentaries
Study type included: cohort, case-control, cross-sectional, and randomized controlled trials	Pediatric studies or studies involving pregnant individuals
Studies must discuss complications of mandibular fracture, and include rates of malunion and nonunion	Lack of full-text availability, duplicate publications, or overlapping datasets

Data Extraction and Reporting

**Figure 1:**
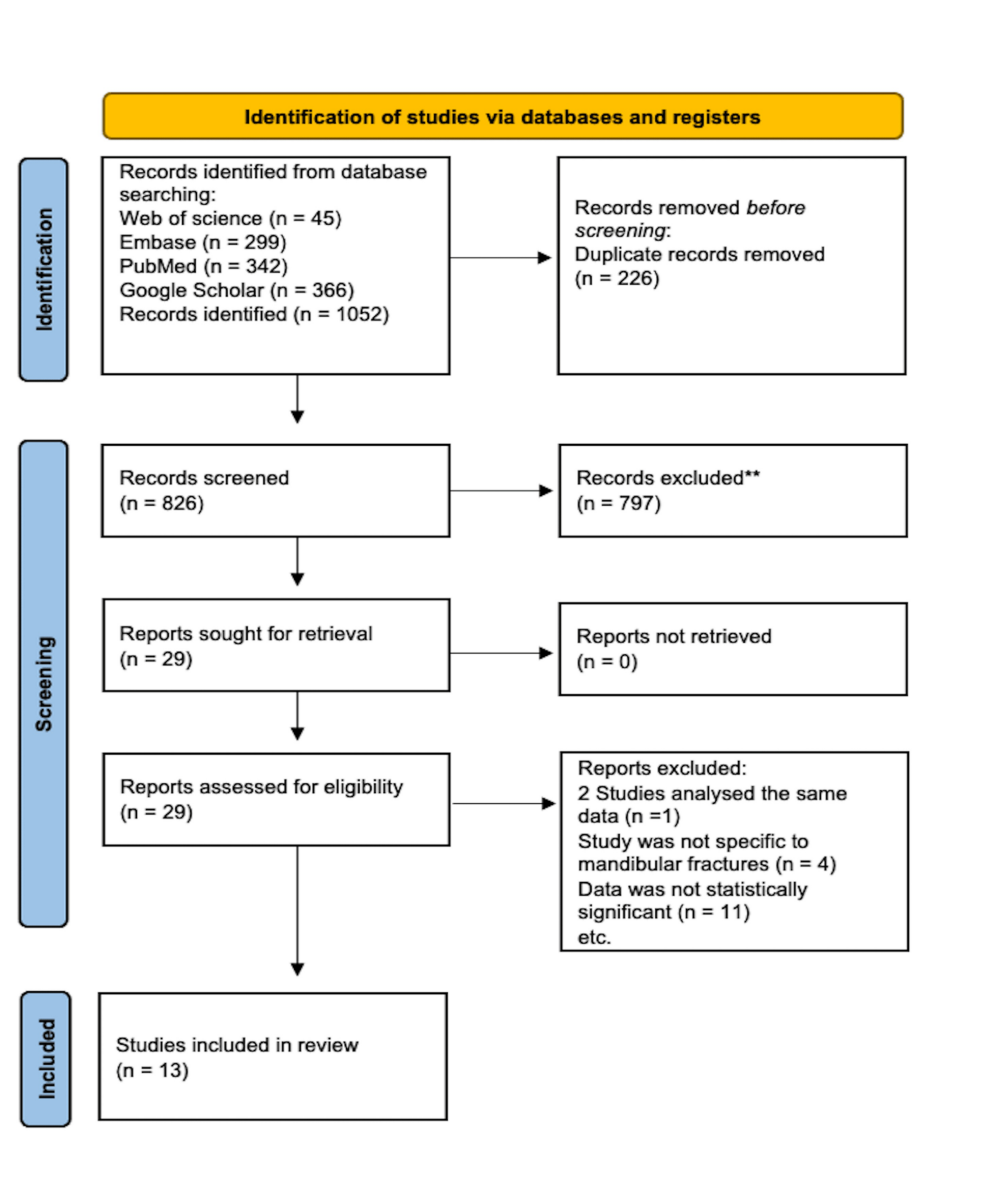
PRISMA diagram Flow diagram created by the authors reflecting the study's selection process in accordance with PRISMA-ScR guidelines [7]. PRISMA-ScR: Preferred Reporting Items for Systematic Reviews and Meta-Analyses - Extension for Scoping Reviews

After the initial data screening, four authors independently performed a secondary screen of 29 articles to determine suitability. All authors then reviewed any discrepancies regarding the inclusion of articles to achieve a consensus of 13 articles for the review. The review utilized the PRISMA Extension for Scoping Reviews (PRISMA-ScR) guidelines, as shown in Figure 1 [7].

Results

The results of the 13 articles chosen for this review are seen in Table 2. Data extracted included the study design, sample size, interventional methods, outcomes of interest relevant to the aim of this scoping review, and a summary of the main findings pertaining to rates of malunion or nonunion.

**Table 2:** Summary of the articles used for this review ORIF, open reduction and internal fixation; MMF, maxillomandibular fixation; CR, closed reduction; IMF, intermaxillary fixation; EPF, external pin fixation; GSW, gunshot wound.

Reference	Study Design	Sample Size	Intervention Type	Relevant Outcome(s) of Interest	Main Findings
Sukegawa et al., 2019 [8]	Retrospective comparative	105	ORIF with reconstruction plate and miniplate	Fracture Characteristics	Type of fracture (both simple and comminuted) has a significant association with malunion
Okoturo et al., 2008 [9]	Randomized comparative	55	Group 1: MMF (closed reduction with arch bars); Group 2: ORIF (Champy’s technique using 2.0 mm miniplates transorally)	CR vs ORIF	MMF reconstruction had a higher rate of malunion compared to ORIF
Sajid et al., 2020 [10]	Comparative clinical	60	Group A: ORIF using reconstruction and/or miniplates; Group B: CR with MMF	CR vs ORIF	CR showed greater rates of nonunion compared to ORIF
Ellis et al., 2003 [11]	Retrospective cohort	196	EPF, CR, ORIF	Fracture characteristics; CR vs ORIF	Patients with comminuted fractures containing ≥5 fragments and fractures due to GSW were associated with higher rates of complications. The ORIF group yielded a better outcome compared to the CR and EPF groups.
Kumar et al., 2018 [12]	Randomized clinical trial	180	ORIF using intraoral or transbuccal approach	ORIF approaches	Patients who underwent the intraoral approach of ORIF had an increased rate of nonunion than those who underwent transbuccal approach
Ansari et al., 2011 [13]	Retrospective observational	53	IMF	Fracture characteristics	Patients with posterior mandible fractures were associated with higher rates of malocclusion, malunion, and nonunion than anterior mandible fractures
Demesh et al., 2019 [14]	Retrospective cohort with biomechanical lab analysis	84	ORIF using metal plating (vertical box plates or traditional rigid internal fixation plates)	ORIF approaches	Vertical box plates were more stable and led to fewer complications compared to linear plating in ORIF
Chen et al., 2018 [15]	Retrospective cohort	135	64.4% of patients had a transcervical approach, 35.6% had transoral treatment	History of substance abuse	Patients who were active smokers at the time of injury experienced increased rate of major complications following ORIF
Wan et al., 2012 [16]	Retrospective cohort	597	ORIF with transbuccal and transoral approaches to insert fixation screws	Age; History of substance abuse; ORIF approaches	Patients >45 years old, smokers, and undergoing ORIF via transoral route had increased incidence of complications
Spinelli et al., 2015 [17]	Retrospective cohort	389	IMF, transbuccal, and intraoral ORIF	History of substance abuse; Fracture characteristics	Patients with alcohol or drug addiction and those with multiple-site involvement were associated with higher complication rates
Lander et al., 2021 [18]	Retrospective cohort	19,152	ORIF	Age; History of substance abuse; Fracture characteristics; Treatment Delay	Patients >65 years old, history of alcohol abuse, open fractures or fractures involving body of mandible, and delay in treatment up to a week increased rates of malunion or nonunion
Hassan et al., 2025 [19]	Retrospective cohort	226	ORIF	Treatment delay	Patients waiting more than 7 days before undergoing ORIF had a significantly higher rate of malunion than those undergoing earlier
Ahmed et al., 2016 [20]	Prospective clinical	60	Nursing teaching protocol covering nutrition, oral hygiene, and jaw exercises	Postoperative care	Patients who received postoperative guidance from nursing staff showed significantly lower rates of malunion and nonunion

Age as a Risk Factor

Two articles discussed older age as a potential risk factor for malunion or nonunion following mandibular fracture. Lander et al. [18] reported that patients above the age of 32 had increased rates of malunion or nonunion, and those over 65 had the greatest odds of a complication. Wan et al. [16] reported that patients aged 45 or older had increased odds of overall complications.

Substance Abuse as a Risk Factor

Four articles demonstrated that previous or current use of tobacco or alcohol abuse was associated with increased postoperative complications, including malunion or nonunion [15-18]. Smoking status was a stronger predictor of surgical outcomes than surgical methods, as seen in one study, which reported significantly higher complication rates in active smokers than in nonsmokers or former smokers [15]. The role of preoperative smoking cessation to improve tissue oxygenation and facilitate postoperative bone healing should not be underestimated. 

Fracture Characteristics as Risk Factors

Five articles discussed fracture characteristics as risk factors for post-treatment malunion or nonunion [8,11,13,17,18]. The characteristics included fracture location, mechanism, and number of fragments. One mechanism discussed was gunshot wounds. These fractures often involve many fragments and are associated with more complications than fractures with fewer fragments. Posterior mandibular fractures are another mechanism discussed, and there are many inherent complications due to the jaw's anatomical features, such as bone structure and muscle attachments. 

Closed Reduction Techniques as Predictors

Three articles discussed closed reduction (CR) techniques as predictors of malunion or nonunion following treatment of mandibular fractures [9-11]. Open techniques were consistently reported to result in fewer postoperative complications, including malunion and nonunion. While ORIF is often preferred, closed reduction techniques such as MMF, as well as hybrid approaches like external pin fixation (EPF), may still be considered in select patients, depending on the fracture's severity and complexity.

ORIF Approaches as Predictors

Three articles provided clinically relevant data regarding malunion and nonunion rates amongst different ORIF approaches [12,14,16]. Vertical box plating and multiple miniplates are plating methods that may lead to preferable clinical outcomes due to increased torsional stability [14]. Inadequate stability is a significant risk factor for malunion and nonunion. With regards to the route of entry, two articles suggest that the transbuccal approach to ORIF may be superior to the intraoral approach due to increased ease of access [12,16].

Treatment Delay as a Predictor

Two articles discussed delays in intervention as predictors for developing postoperative malunion or nonunion [18,19]. Both articles suggested that a delay in treatment of more than a week may lead to more postoperative complications in patients. Decision-making can be complicated, as operating on an acutely inflamed mandible may increase the risk of wound dehiscence. The timing of surgical intervention to balance unnecessary delay with non-ideal operative conditions may prove to be a challenge and must be approached cautiously.

Postoperative Care as a Predictor

One article discussed the effect of postoperative patient education on complication rates [20]. Patients were randomly assigned to groups to receive routine hospital care or a suggested nursing teaching protocol. Nursing staff successfully guided patients on nutrition, oral hygiene, and exercise to reduce postoperative complications. Increased emphasis on patient education was associated with better outcomes.

Discussion

Age as a Risk Factor

Older age was found to be a potential risk factor for malunion or nonunion following mandibular fracture. Lander et al. analyzed the demographics of 161 patients who developed malunion or nonunion following ORIF for isolated mandibular fractures [18]. Compared with patients aged 18-20 years (n < 11), those aged ≥32 years had a more than five-fold increased risk of malunion or nonunion after adjustment for comorbidities and fracture characteristics. Patients aged 32-44 years (n = 52), 45-65 years (n = 38), and ≥65 years (n < 11) had significantly increased odds of malunion or nonunion, with adjusted odds ratios of 5.44, 5.20, and 5.47, respectively (all p < .05). Wan et al. also found that older age was associated with increased risk of postoperative complications including malunion and nonunion. Out of all patients with complications following ORIF (n = 82), patients over the age of 45 were at increased odds of complications compared to those aged <35 (aOR, 1.98; p = .045). Advanced age may be associated with increased risk for postoperative malunion and nonunion, likely due to propensity for falls, diminished bone quality, and inadequate dentition.

History of Substance Abuse as a Risk Factor

Previous or current use of substances, specifically tobacco or alcohol abuse, was demonstrated to have an association with increased postoperative complications, including malunion or nonunion. Wan et al. found that smokers (n = 379) had increased odds of complications, including malunion and nonunion, compared to nonsmokers (n = 268) (OR, 1.72; p = .047) [16]. Chen et al. found that compared with patients who had never smoked or were former smokers (n = 37), active smokers (n = 66) were at increased odds of developing major complications, including malunion or nonunion (OR, 4.04; p = .04) [15]. Spinelli et al. investigated the rates of postoperative complications after patients underwent ORIF with transbuccal miniplate fixation. The study produced statistically significant data that showed patients with alcohol or drug addiction (n = 180) had higher rates of complications (p < .05), including nonunion, than those who did not abuse substances (n = 209) [17].

Lander et al. reported that patients who abused alcohol (n = 2993) had increased odds of developing malunion or nonunion following mandibular fracture (aOR, 1.61; p < .05) [18]. Overall, patients who abuse alcohol or other substances are approximately 1.6 to 4.0 times as likely to experience postoperative complications, including malunion or nonunion. Alcohol and substance abuse are patient-specific factors that must be considered in determining the risk of complications before surgical intervention to ensure postoperative care and follow-up are conducted accordingly.

Fracture Characteristics as Risk Factors

Fracture characteristics are pertinent risk factors for post-treatment malunion or nonunion, including fracture location, mechanism, and number of fragments. Ellis et al. looked at 196 patients with differing complexities of fractures of the mandible and compared different approaches to correction. The overall incidence of nonunion was 3.5% (n = 7), occurring only in patients with highly comminuted fractures containing five or more fracture fragments. The degree of fragmentation and rates of nonocclusal complications were shown to be associated with the mechanism of injury (p <0.05), with the greatest occurrence of nonunion seen in gunshot wounds (GSW). Of all participants who suffered a GSW involving the mandible, 22.2% developed a nonocclusal complication such as nonunion [11].

Ansari et al. evaluated anterior and posterior mandible fractures treated with intermaxillary fixation (IMF) bone screws. In patients who suffered a posterior mandible fracture (n = 32), 15% suffered from either malunion, malocclusion, or nonunion. Of the participants with anterior fractures (n = 21), 0% experienced any type of occlusal complication. Patients with anterior mandible fractures had a significantly lower rate of malocclusion or malunion than those with posterior mandible fractures (p < .001), suggesting a relationship between fracture location and the risk of improper union [13]. Spinelli et al found that patients with multiple site involvement (n = 141) were associated with a higher rate of complications, including nonunion and malunion, compared to those with isolated fractures (n = 248) (p < .05) [17].

Lander et al. found that patients with open fractures (n = 41) or fractures involving the body of the mandible (n = 51) were associated with nearly twice the odds of malunion or nonunion, with adjusted odds ratios of 1.72 and 1.73, respectively (both p < .05), compared to closed fractures. Contrastingly, patients with fractures of the subcondylar region had about half the odds of enduring impaired healing (aOR, 0.57; p < .05) [18]. Sukegawa et al. also found a significant association between fracture type (simple and comminuted) and incidence of malunion (p = 0.023) [8].

Ultimately, the five articles provide evidence to support the importance of recognizing fracture type, location, or mechanism as potential risk factors for post-treatment malunion or nonunion. Overall, it appears open, multiple-site fractures and fractures of the body of the mandible demonstrate the greatest risk for the development of malunion and nonunion.

CR vs ORIF as Predictors of Complications

In a study by Okoturo et al., rates of malunion were compared between patients undergoing MMF (closed reduction with arch bars) and open reduction. The MMF group (n = 30) had a malunion rate of 13.3%, while the open reduction group (n = 25) had a rate of 0% (p < .05) [9]. Sajid et al. found a decreased rate of nonunion in ORIF patients (n = 30) when compared to closed reduction patients (n = 30) for comminuted mandibular fractures. The study observed a nonunion rate of 6.7% in patients treated with CR, while none of the patients that underwent ORIF developed nonunion (p < .03) [10].

Ellis et al. looked at 196 patients with comminuted fractures of the mandible, and compared three different approach methods: ORIF (n = 146), CR (n = 35), and EPF (n = 17). The study showed that the treatment approach was significantly associated with the incidence of nonocclusive complications, including nonunion. The overall rates of nonocclusive complications for EPF, CR, and ORIF were 23.5%, 17.1%, and 5.5%, respectively (p < .01) [11]. The aforementioned studies suggest that open approaches may be preferable to closed techniques for reducing the risk of malunion or nonunion following mandibular fractures.

ORIF Approaches

Open reduction and internal fixation of mandibular fractures is performed via various approaches with respect to the plating method and entry approach. Demesh et al. compared rates of complications overall, including nonunion, between vertical box plates and linear plating. The vertical box plate group (n = 18) had an overall complication rate of 6%, compared to 41% in the traditional plate group (n = 66) (p = 0.03) [14]. In a randomized controlled trial, Kumar et al. compared the complication rates between intraoral and transbuccal approaches for ORIF. A group of 180 patients with bilateral mandible angle fractures was split into two groups of 90, intraoral and transbuccal, respectively. In the intraoral group, 11.1% of patients developed nonunion, whereas in the transbuccal group, the incidence was 1.1% (p = 0.005) [12]. Similarly, Wan et al. found that the odds of complications, including nonunion or malunion, increased 1.71 times in the transoral group (n = 370) when compared to patients who underwent a transbuccal technique (n = 227) (p = .04) [16]. While the transbuccal approach seems to yield better outcomes, surgeon preferences and fracture characteristics may play a role in decision-making.

Treatment Delay as a Predictor

Delays in intervention as predictors for developing postoperative malunion or nonunion were correlated with increased negative outcomes of fracture healing. Hassan et al. compared patients who waited more than seven days before undergoing ORIF (n = 24) to patients who had the procedure done within three days (n = 168). The rate of malunion for the group that waited >7 days to have the procedure and the group that waited <4 days was 12.5% and 2.4%, respectively (p = 0.043) [19]. Lander et al. also explored the effect of treatment delay. Compared with patients who had same-day treatment (n = 23), patients who waited six to seven days to get treatment (n = 14) had almost twice the odds of malunion or nonunion (aOR 1.84; p < .05) [18]. These studies show the importance of prompt surgical intervention in avoiding postoperative malunion and nonunion.

Postoperative Care as a Predictor

Lack of postoperative patient education may be predictive of increased malunion rates. Ahmed et al. revealed important findings regarding an enhanced postoperative nurse teaching protocol, involving oral care, nutrition, and jaw exercises. Patients receiving comprehensive postoperative instructions and guidance experienced a significant reduction in rates of malunion following mandibular fractures (p < .038). Two weeks after discharge, the enhanced nursing protocol group (n = 30) had no cases of malunion. On the other hand, the normal hospital care group (n = 30) had a malunion rate of 13.3%. The study indicates that a robust postoperative management protocol is crucial to reducing rates of nonunion and malunion by promoting behaviors that facilitate healing [20].

Clinical implications and recommendations

The analysis of current research demonstrates the importance of providers recognizing patient risk factors and choosing optimal intervention strategies to reduce rates of postoperative malunion and nonunion following mandibular fractures. Based on this review's findings, patient history may contribute to a complicated postoperative course. Generally, patients above 45 years of age and patients with a history of drug or alcohol abuse should be followed more closely to ensure appropriate healing ensues. Additionally, comminuted mandibular fractures, fractures involving multiple sites, and GSW victims should be handled with caution. A thorough assessment of injury extent and fracture nature may improve preoperative decision-making and postoperative outcomes.

Overall, ORIF was superior to all other intervention types, including CR and EPF, in reducing the incidence of malunion or nonunion. Choice of intervention should be based on patient-specific needs; however, it is important to note that certain fixation methods associated with ORIF may provide improved mechanical stability and enable an environment suitable for proper bone reunion. Additionally, unnecessary delays in treatment should be avoided to reduce the risk of hindered recovery.

Limitations and future directions

This review reflects certain limitations that must be considered when interpreting the results. Many studies grouped malunion and nonunion with other postoperative complications, such as malocclusion and infection, in their statistical analyses. The nonspecific associations between risk factors or predictors and other complications may serve as potential confounders when attempting to draw conclusions about bone reunion alone. This is likely due to the rarity of the occurrence and the lack of sufficient data relevant to this topic in the current literature. Another limitation was the lack of consistency in reporting of fracture characteristics when assessing the impact of treatment modality on postoperative malunion and nonunion. Additionally, inconsistent follow-up intervals, especially for nonunion cases, may have led to complication rates being underestimated.

Future investigations should emphasize the role of patient education and postoperative care in the development of malunion or nonunion following mandibular fracture treatment. It is important to establish guidelines to direct treatment based on fracture mechanisms and other fracture characteristics, such as fragmentation, to standardize the approach regardless of surgeon preference. While malocclusion has been well documented, a deeper exploration of factors affecting malunion and nonunion may lead to better postoperative outcomes and reduce reoperation rates.

## Conclusions

This review highlights the multifaceted approach required when assessing and managing mandibular fractures. Exploring patient risk factors such as age, substance abuse, and fracture complexity is key for outcome improvement; the aforementioned factors have all been linked to higher rates of malunion and nonunion. Prompt intervention and postoperative patient education protocols should be a priority, as delays in treatment, along with insufficient postoperative guidance, have both been shown to increase rates of complications. Additionally, recognizing the success rates of ORIF techniques while appreciating the nuances of each treatment modality will enable clinicians to provide optimal care specific to patient needs.

## Appendices

Database Queries

**Table 3:** Database Queries Table reflecting individualized search queries based on Web of Science, Embase, Pubmed, and Google Scholar search mechanics.

Web of Science - *09/28/2025*		
1	ALL=(Mandibular fracture OR "mandible fracture" OR "jaw fracture" OR "maxillofacial fracture")	8,475
2	ALL=(Surgery OR "surgical management" OR "operative procedures" OR "open reduction" OR ORIF OR "internal fixation" OR “closed reduction)	4,409,322
3	ALL=(malunion OR "fracture malunion" OR nonunion OR "bone healing complications" OR "postoperative complications")	102,055
4	ALL=(malocclusion OR "dental occlusion" OR "occlusal complication" OR "occlusal dysfunction")	14,627
5	#3 OR #4	116,530
6	#1 AND #2	5,958
7	#5 AND #6	740
8	#7 AND ALL=(human)	45
Embase - *09/28/2025*		
1	'mandible fracture'/exp OR mandibular fracture*:ti,ab OR mandible fracture*:ti,ab	13,719
2	'internal fixation of fracture'/exp OR ORIF:ti,ab OR 'open reduction':ti,ab OR 'surgical management':ti,ab	101,573
3	'malocclusion'/exp OR malocclusion:ti,ab OR 'postoperative malocclusion':ti,ab OR malunion:ti,ab OR 'fracture malunion':ti,ab	48,223
4	#1 AND #2 AND #3	303
5	#4 AND 'human'/de	299
PubMed - *09/28/2025*		
1	"Mandibular Fractures"[Mesh] OR mandibular fracture*[tiab] OR mandible fracture*[tiab] OR jaw fracture*[tiab]	4,741
2	"Fracture Fixation, Internal"[Mesh] OR "Surgical Procedures, Operative"[Mesh] OR surgery[tiab] OR surgical management[tiab] OR operative fixation[tiab] OR "open reduction"[tiab] OR ORIF[tiab]	2,864,010
3	"Fracture Healing"[Mesh] OR malunion[tiab] OR "fracture malunion"[tiab] OR nonunion[tiab] OR "healing complication*"[tiab] OR "postoperative complication*"[tiab]	120,138
4	"Malocclusion"[Mesh] OR malocclusion[tiab] OR "dental occlusion"[tiab] OR occlusal complication*[tiab] OR occlusal dysfunction[tiab]	19,175
5	#3 OR #4	139,032
6	#1 AND #2 AND #4	405
7	#5 AND #6	405
8	#7 AND humans[Mesh]	342
Google Scholar - *09/28/2025*		
1	intitle:"mandibular fracture" OR intitle:"mandible fracture" AND (ORIF OR "open reduction" OR "internal fixation") AND ("postoperative malocclusion" OR "postoperative malunion" OR "fracture malunion" OR nonunion)	366
